# Demographic dynamics of waterborne disease and perceived associated WASH factors in Bushenyi and Sheema districts of South-Western Uganda

**DOI:** 10.1007/s10661-023-11270-1

**Published:** 2023-06-20

**Authors:** Hope Onohuean, Uchechukwu U. Nwodo

**Affiliations:** 1 Biopharmaceutics Unit, Department of Pharmacology and Toxicology, School of Pharmacy, Kampala International University Western Campus, Ishaka-Bushenyi, Uganda; 2 Patho-Biocatalysis Group (PBG), Department of Biochemistry and Microbiology, University of Fort Hare, Private Bag 1314, Alice, 5700 Eastern Cape South Africa

**Keywords:** Demographic attributes, Perceived, WASH, Waterborne disease, Intervention, Uganda

## Abstract

Water remains a significant player in spreading pathogens, including those associated with neglected tropical diseases. The implications of socio-demographic delineations of water quality, sanitation, and hygiene (“WASH”) interventions are on the downswing. This study assessed waterborne diseases and perceived associated WASH factors in the Bushenyi and Sheema districts of South-Western Uganda. This study examines the linear relationship between WASH and identifies the association of specific demographic factors as well as their contributions/correlations to waterborne disease in the study area. A structured qualitative and quantitative data collection approach was adopted in face-to-face questionnaire-guided interviews of 200 respondents on eight surface water usage. Most participants, 65.5%, were females and had a higher score of knowledge of WASH (71%), 68% score on the improper practice of WASH, and 64% score on unsafe water quality. Low score for basic economic status was (57%), report of common diarrhoea was (47%), and a low incidence of waterborne disease outbreaks (27%). The principal component analysis (PCA) depicts the knowledge and practice of WASH to have a strong positive correlation (*r* = 0.84, *p* < 0.001; *r* = 0.82, *p* < 0.001); also economic status positively correlated with grade of water source, knowledge, and practice of WASH (correlation coefficient = 0.72; 0.99; 0.76 and *p*-values = 0.001; < 0.001; < 0.001 respectively). Occupation (*p* = 0.0001, *OR* = 6.798) was significantly associated with knowledge and practice of WASH, while age (*r* = −0.21, *p* < 0.001) was negatively associated with knowledge and practice of WASH. The basic economic status explains why “low economic population groups” in the remote villages may not effectively implement WASH, and diarrhoea was common among the population. Diarrhoea associated with unsafe water quality and improper practice of WASH is common among the study population, and there is a low incidence of waterborne disease outbreaks. Therefore, government, stakeholders, and non-governmental organisations should work together to promote proper practice of WASH conditions to limit the occurrence of diarrhoea and prevent potential waterborne disease outbreaks.

**Supplementary Information:**

The online version contains supplementary material available at 10.1007/s10661-023-11270-1.

## Background

Water is life and one of the most valuable natural resources that sustain lives, while contamination of its sources is a problem to the community. Access to quality and potable water supply is a public health issue, especially among the population living in remote villages and hard-to-reach communities in mid and low-income countries (Onohuean et al., 2021b). Water quality, sanitation, and hygiene (WASH) is a key factor in international development to achieving Sustainable Development Goal 6 (SDG 6). World Health Organization (WHO) and United Nations International Children’s Emergency Fund (UNICEF) report of 2017 estimates that 2.5 billion (35% of the world population) lack proper or improved sanitation facilities (Pruss-Ustan et al., 2008; DeNavas-Walt & Proctor, 2014; Adams et al., 2016; Fanucchi, 2016; United Nations, 2019), and 844 million people lack access to quality and portable drinking water (Assembly, 2019; Fanucchi, 2016). Also, another report has shown an annual death of 700,000 children, and many are living with ill-health conditions, poor physical health, and cognitive development in developing countries (Haller & Guy Hutton, 2007). Globally, about 159 million people drink untreated surface water sourced from streams, running waters, wells, lakes, or rivers (WHO & UNICEF, 2017). Additionally, more than a third of the world’s population lacks basic sanitation and proper disposal of human waste (Centre for Diseases Control and Prevention, 2015), while surprisingly, a solitary 19% of people wash their hands with soap after coming into touch with excreta (Korber et al., 2020). The 801,000 children (< 5) who die from diarrhoea each year, 88%, are associated with diarrhoeagenic illnesses due to unsafe drinking water, poor hygiene, and inadequate sanitation practices (Pruss-Ustan et al., 2008) (Liu et al., 2012).

In 2015, faecal water contamination caused about 1.3 million deaths (Troeger et al., 2017). Most of the neglected tropical diseases (NTDs), schistosomiasis, Guinea worm disease, Buruli ulcer, trachoma (Hotez et al., 2006; World Health Organization, 2009), and *Vibrio* cholera (Onohuean et al., 2021a, b), which affect millions of people worldwide, are mainly distributed in the water. Also, the soil-transmitted helminth infection that infects 1 billion people (Jourdan et al., 2018; WHO, 2018) is attributed to poor hygiene practices, inadequate sanitation, and unsafe drinking water. Poor or lack of water quality, sanitation, and hygiene (WASH) facilities is the primary cause of infant illnesses and have deprived many children of education, burdening mothers and reducing work productivity (UN, 2012, 2015). In contrast, the impact of water quality, knowledge, and practice of WASH is under-reported in resource-limited settings. Thus, our study is anchored on the hygiene improvement framework of the EHP/UNICEF/WES/USAID (2004) protocol (EHP/UNICEF/WES/USAID, 2004).

Sub-Saharan Africa is one of the regions classified in 2006 by WHO and UNICEF with the most deficient coverage of improved sanitation of 31%, Southern Asia (33%), and Eastern Asia (WHO, 2013). In Uganda, one the sub-Saharan nations, “poor sanitation and hygiene, as well as unequal access to safe drinking water, make thousands of children very sick and at risk of death” (MWE, 2015; Nayebare et al., 2020), while about a tenth of the population defecates in the open, and two thirds of the homes do not wash their hands with soap. Diarrhoea is one of Uganda’s three greatest childhood killers, with a daily fatality rate of 33 children (UN, 2015; WASH-Uganda, 2017). Children are frequently affected by drinking polluted water or coming into contact with faecally contaminated water, such as playing or swimming in a contaminated pool and open drains, or contaminated hands from parents or caregivers who may not have washed their hands with soap or a specific disinfectant. There are reports on water-related diseases from remote villages of greater Bushenyi districts in Uganda by health centre IV and mainstream research findings but have limited implementation pace and translation into meaningful intervention (Onohuean et al., 2021a, b; Paul, 2018). Findings from primary health data may also impact the region’s mortality and morbidity ratio of the current disease state. However, these are not commonly made public due to health systems management issues and ethics, preventing the needed help such reports would have had on the people and minimising the few evidence-based interventions and necessary impact of such studies. Most local and remote Uganda villages and communities have no regular surveillance systems, and some information reported in the district health information system lacks practical research details. Most of them only consisted of preliminary data for health planners to glimpse local health data and lack future strategic development agendas. These, therefore, warrant organised investigation to establish verifiable data for interventions that may assist in making lives better for the rural communities.

Most common studies for identifying sociocultural context-specific characteristics in public health interventions and limiting outbreak are knowledge, attitude, and practice (KAP) surveys (Werner, 1977). Understanding the socio-demographic and economic facets of the context in which these treatments are implemented is crucial for the success of public health interventions (PHIs) in resource-constrained settings (WHO-TB Partenership, 2008). However, demographic changes and economic status inevitably influence societal beliefs and practices; hence, it is crucial to take these changes into account while developing PHIs (Raihan et al., 2019). To the best of our knowledge, there is no study on WASH-related interventions in Bushenyi and Sheema districts focusing on relationship between WASH variables and the potential influence of demographic factors and basic economic traits. This study sough to define the linear relationship between water quality, sanitation, and hygiene (WASH) and identifies the association of specific demographic factors and basic economic status as well as their contributions/correlations to waterborne disease in the study area.

## Methodology

### Study design and study period

This community-based cross-sectional study used both qualitative and quantitative data extraction method to analyse perceived water quality, sanitation, and hygiene between February and May 2019 in the Bushenyi and Sheema districts of South-Western Uganda.

### Study site and study area

**Fig. 1:**
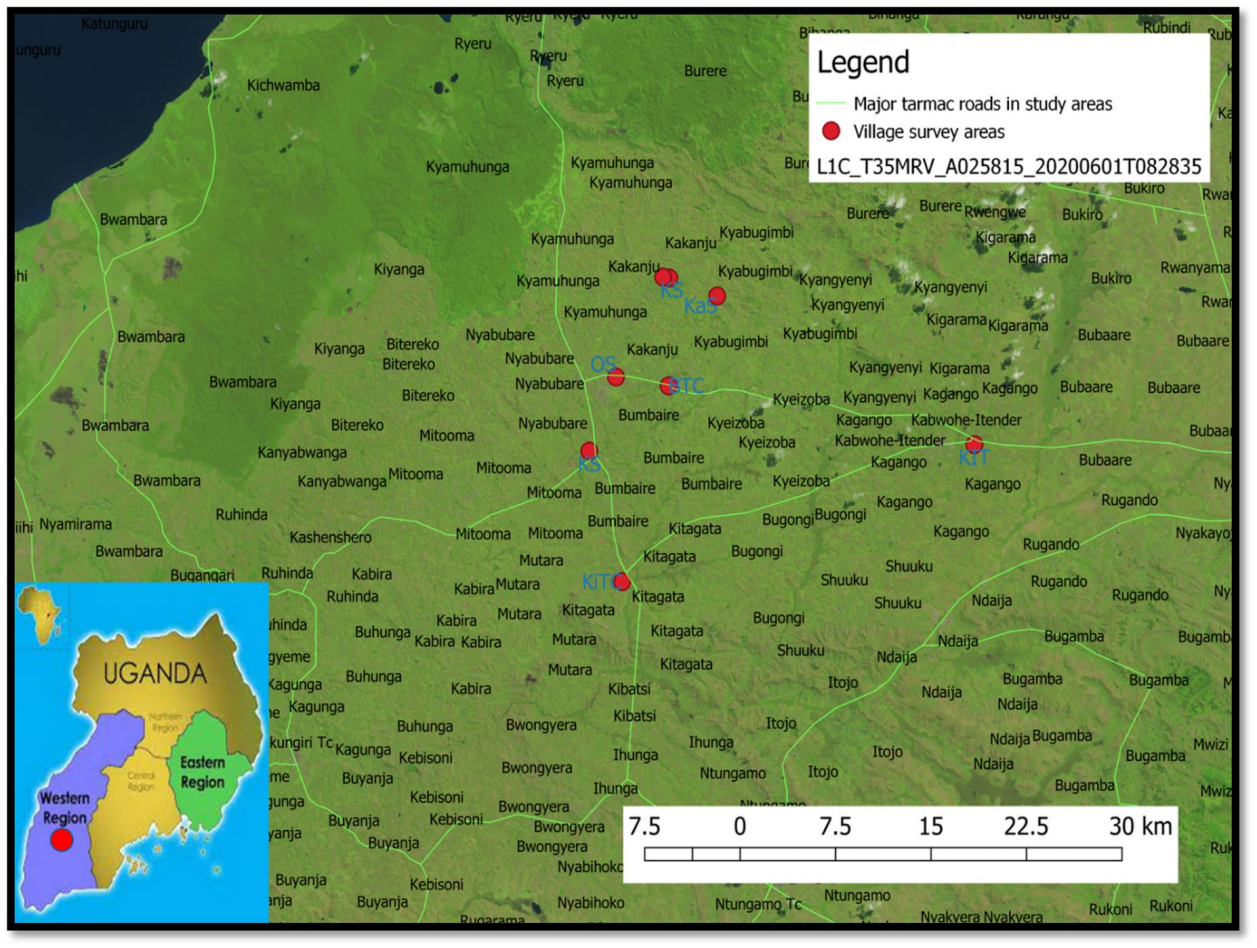
Map of the study region in Uganda. On the left is the map of Uganda showing the study region’s location, while the right shows the various points where the questionnaire was administered with a face-to-face interview of respondents. BTC, Bushenyi Town council; KS, Kashenyi spring; KaS, Kagogo spring; KL, Katungu Lake; KS, Katungu Spring; OS, Orushenyi spring; KIT, Kabwohe Itender town council; KiTC, Kitagata Town Council

The study sites include the following: Kabwohe natural raw water from Katagata mountainous spring and Kitagata natural raw water from Swamp sub-county of the Sheema district; Nyamizinga Bushenyi natural raw water and Rwamuro Nyabukurunga natural raw water both from Swamp; and Katunga tap water, Katunga Lake, Katunga spring, and Orushenyi-Ishaka open spring water of Bushenyi district are in the Western region of Uganda, East Africa (Fig. 1).

The Bushenyi district is spread across Kabwohe in the West, Buhweju in the south, Rubirizi in the East, and Mitooma in the North, covering 590 km. Bushenyi district has an estimated population of 251,400, and Ishaka has been the most significant town according to the 2012 national population census, with the economy mainly dependent on agriculture (Statistics, 2017; UBOS, 2017). The majority of the population practice subsistence farming, while few are involved in the commercial production of coffee, sweet bananas, tea, and matooke. Furthermore, there is widespread dairy farming and ranching for beef aimed at subsistence and commercial purposes. Sheema district’s former Sheema county under Bushenyi district before becoming operational in 2010 has a population estimate of 220,200 (Statistics, 2017; UBOS, 2017). The majority of the population grew crops and production of livestock for subsistence and commercial purposes.

Map of the study area was plotted using settings in qGIS^®^, an open-source, free software. The Sentinel-2 image ID: L1C_T35MRV_A025815_20200601T082835 dated 7 September 2020 was obtained from the United States Geographical Surveys (USGS) and superimposed on a shapefile for Uganda and roads. + e satellite image file was modified to show land vegetations.

### Sample size determination

Eight identified surface water locations and the population of persons living within the geographical region of the study location were used for the study. According to the diarrhoea infections report of Bushenyi health centre IV, the questionnaire was administered to 200 study participants within the study population attributed to water-related illness in the communities detailed in (S3) supplementary material.

### Questionnaire design and quantitative data collection

A structured questionnaire prepared based on reviewed relevant literature in the English language was used to collate perceived WASH data from key respondent informants. The questionnaire comprises a total of 41 questions. Only 29 questions were analysed, categorised as follows: 5 questions about water quality, four questions on knowledge of WASH, six questions on steps in the practice of WASH, six questions to evaluate the basic economic status of the study population, and one question each on the incidence of waterborne disease and intervention respectively. The questionnaire covered seven study participant demographics (S1 in Supplementary Material). Study participants that qualify with the inclusion criteria were recruited and interviewed with the questionnaire, and responses were collated adequately.

### Qualitative data collection

We used the content of the questions to thematically collect qualitative data from key informant by interview and investigator team discussions.

### Quality control

The questionnaire was pretested to KIU School of Pharmacy students and Basaja Market women in Bushenyi district for data collection completeness, reliability, and consistency from field reports. Their detailed responses were noted, and the questionnaire was updated/edited/corrected based on questions, respondents’ comments, easy understanding, data collectors, and supervision a week before data collection. Four research team members (one postdoc microbiologist and epidemiologist, one biochemistry pre-doctoral student, one MSc epidemiology, and one BSc laboratory scientist) collected the data; a supervisor and a luganda/banyankore interpreter were trained/recruited a day before collection on the aim and objectives of the study and method of approaching the study participants to enhance the recoverability of the questionnaire from the study participants.

### Defining context

- WASH: This is an acronym for “water quality, sanitation, and hygiene”. It emphasises water sources, sanitation, and hygiene study population’s actions toward an open-defecation-free environment, hand-washing, and keeping the region’s drinking water safe.
- Water quality: Water quality describes the perceived condition of the water, including chemical, physical, and biological characteristics, usually concerning its suitability for a particular purpose such as drinking and general household uses.
- Knowledge of WASH: facts, information, and skills acquired through experience or education as it relates to WASH.
- Practice of WASH: the actual application or use of an idea, belief, or method, as opposed to theories relating to WASH.
- Basic economic status: the fundamental social standing or class of an individual in the study population according to the Uganda Bureau of Statistics 2020.
- Incidence of waterborne disease: the measure of disease that allows us to determine a person’s probability of being diagnosed with a disease due to unsafe water, sanitation, and hygiene.
- Intervention: The action taken to improve diseases due to unsafe water, sanitation, and hygiene.

### Ethical considerations

This study received ethical clearance from the ethical committee of Kampala International University (KIU), Western campus, Ishaka-Bushenyi Uganda (UG-REC-023/201919). A formal letter was written to District Health Office, Bushenyi Local Government, for concern and approval. Oral communication, ethical clearance, and personal identification were shown to the local government chairperson (LC1) for communities’ entrance to assess the surface waters in each community. A word-of-mouth/consent letter to seek the consent of each study participants at the site of question administration (S2 text). The study’s goal was made clear to the study participants /respondents and allowed to agree with the interview process. The confidentiality of the study participants was secured by not taking any form of identification of the study respondents.

#### Inclusion criteria

Study participants who voluntarily agreed to participate and are residents within a 200-m radius/circumference away from the surface water locations were recruited and included in the study.

#### Exclusion criteria

Visitors, recreational explorers, and individuals who lived more than 200 m away from the surface waters were excluded.

### Data analysis

Data were entered into the Microsoft Excel program and cleaned and analysed using R and IBM SPSS version 20 for descriptive statistics, percentages, and mean values. The principal component analysis (PCA) and Pearson correlation analysis were used to evaluate the relationship between knowledge and practice of the WASH matrix with selected demographics, also the same as the relationship between water quality, economic status, and incidence of disease and interventions. A further multivariate logistic regression model was used to identify the demographic factors associated with knowledge and practice of WASH. The significance of the association is presented in an odds ratio with 95% CI and *p*-value.

### Quantitative data

We develop the water quality, knowledge of WASH, the practice of WASH, and basic economic status metrics. The study participants’ water sources were graded as water quality into safe or unsafe, and answers to the questions in each section (S1 in Supplementary Material) were graded as follows: knowledge of WASH into good or bad, the practice of WASH into proper or improper, and basic economic status into stable or unstable. To make the water quality metric (WQ_Matrics), the proportion of safe or unsafe responses to the questions for each participant was transformed into a binary outcome, i.e., suitable quality (WQ_Matrics > 50%) and insufficient quality (WQ_Matrics < 50%) used for the logistic regression model. The same approach was employed to develop metrics for knowledge of WASH, the practice of WASH and basic economic status. The contribution of each question to these metrics was then evaluated and employed in the principal component analysis. The correlation coefficient with the component that described the most appropriate variation was used to calculate the contribution weight for each question.

### Principal component analysis of variables

To explore the relationships between WASH variables, questions in each variable were graded into metrics, {safe/unsafe or (1/2) for water quality (WQ_matrics), good/bad or (1/2) for knowledge of WASH (K_matrics), proper/improper or (1/2) for the practice of WASH (P_matrics), stable/unstable or (1/2) for basic economic status (ECO_STAT)}, and used to evaluate the linear relationship between the WASH parameters. The correlation was described by: (a) visualising the variability of the 200 data points sideways four orthogonal lines corresponding to four component variables and (b) calculating and comparing the correlation coefficients between WASH factors and the demographics parameters.

WASH parameters with a direct relationship show a positive correlation coefficient, while those with opposite relationships indicate a negative correlation coefficient. We used Cronbach’s alpha (Santos, 1999; Gliem & Gliem, 2003; Santos & Reynaldo, 2013) (Table 3) to ensure and validate relatively consistent and reliable factors included in the principal component analysis used in linear relationships.

### Analysis of relationship associations between knowledge of WASH, practices of WASH, and selected study participant demographics by logistic regression

A multivariable logistic regression model was explored using the demographic outcome variable to identify variables that could predict suitable water quality. Each participant’s demographics and responses to questions on knowledge of WASH, Practices of WASH, and basic economic status were evaluated. Initially, the relationship of each variable to the outcome was compared by univariable analysis while the odds ratios, *p*-values, and confidence intervals (CIs) are presented in tables. Variables with a *p*-value < 0.25 were then used to develop a logistic regression model.

### Determination of qualitative data

Qualitative data were analysed using questions content thematic tactic according to Graneheim and Lundman’s report (Graneheim & Lundman, 2004). The study theme was identified and transliterated into patterns that addressed the study’s objectives.

## Results

### Demographics of study participants

The demographic observation of participants shows that the significant study participants were female, 65.5% (131/200), and about 65.0% (130/200) were married, as presented in Table 1. Fifty-two percent (104/200) are peasant farmers as sources of livelihood, 49.5% of age respondents range from 19 to 30 years, and 47.0% (94/200) had only primary education. Individual responses range from 28 to 23 (14.0 to 11.5%) in the eight sites. Although there is a low incidence of waterborne disease outbreaks in the study villages 3% (6/200), there is a high rate of watery diarrhoea, 47% (94/200), and the univariable analysis of these relationships is shown in Table 2.

**Table 1:** Informant demographics

**Features**		**Frequency**	**Percent**
**Age of study participants**	18 years and below	18	9.0
	19–30 years	99	49.5
	31–42 years	46	23.0
	43 and above	37	18.5
**Sex of study participants**	Female	131	65.5
	Male	69	34.5
**Level of education**	Degree	8	4.0
	O level	84	42
	Primary	94	47.0
	No education	14	7.0
**Occupation of study participants**	Student	29	14.5
	Peasant	104	52.0
	Business	25	12.5
	Others	42	21.0
**Marital Status**	Married	130	65.0
	Single	63	31.5
	Widow	7	3.5
**To evaluate risk of waterborne disease and interventions**			
Have there be any incidence of outbreak of water borne disease in this village?	Yes	6	3
	No	194	97
Have there be any incidence of these related water borne disease symptoms in this village?	Cholerae	49	24.5
	Watery diarrhoea	94	47
	Watery diarrhoea + vomiting	21	10.5
	Watery diarrhoea + stomach pains	36	18
Have there be any interventions?	Yes	44	22
	No	156	78

**Table 2:** The univariable relationship between knowledge matrix and demographics, water quality, basic economic status, practices of WASH, and waterborne-related disease

**Category**	**Questions**	**Response**	**Odds ratio (95% confidence interval)**	***p*-value**
Demographic	Sex	Female	Ref	
		Male	0.28 (2.38, 5.45)	0.1
	Age	Under 18	Ref	
		19–30 years	9.91 (0.43, 0.56)	0.00
		31–42 years	3.02 (0.18, 0.29)	0.00
		43 and above	2.29 (0.14, 0.24)	0.016
	Education	O level	Ref	
		Primary	1.23 (0.40, 0.54)	0.68
		No education	0.10 (0.04, 0.11)	0.00
		Degree	0.06 (0.02, 0.07)	0.00
	Occupation	Student	Ref	
		Peasant	6.39 (0.45, 0.59)	0
		Others	0.84 (0.08, 0.18)	0.91
		Business	1.57 (0.16, 0.27)	0.24
	Marital status	Married	Ref	
		Single	0.25 (0.25, 0.38)	0.00
		Widow	0.02 (0.02, 0.07)	0.00
	Districts	Sheema	Ref	
		Bushenyi	0.13 (4.93, 12.30)	0.22
Water quality (WQ)	What is the main source of water for the village?	Open spring/well	Ref	
		Ponds/GRW	0.26 (0.09, 0.19)	0.00
		Lake	0.75 (0.25, 0.38)	0.43
		Tap/borehole water	0.37 (0.13, 0.24)	0.00
	Which of these sources of water do you use?	All	Ref	
		Lake only	2.68 (0.23, 0.36)	0.00
		Open springs only	4.36 (0.34, 0.47)	0.00
		Tap/borehole	1.27 (0.12, 0.22)	0.79
	How do you fetch the water?	Bucket/bowl	Ref	
		Jerrycan	0.02 (27.01, 88.81)	0.56
	Do you treat your water before drinking?	No	Ref	
		Yes	12.57 (0.05, 0.13)	0.31
	If yes what type of water treatment is common in the village?	No response	Ref	
		Boiling	10.57 (0.06, 0.14)	0.21
	What is the mode of storage of water in the village?	Drum/tank	Ref	
		Jerrycan	0.007561 (63.95, 273.38)	0.71
Basic economic status (ECO_STAT)	What is your current income per month?	Below 50,000	Ref	
		60,000–100,000	1.13 (0.25, 0.38)	0.93
		100,000–500,000	0.71 (0.17, 0.28)	0.35
		Above 500,000	0.57 (0.14, 0.24)	0.05
	Do you own a house, land?	No	Ref	
		Yes	2.25 (0.30, 0.66)	0.04
	Do you own radio, television?	No	Ref	
		Yes	1.56 (0.43, 0.95)	0.01
	How many people stay in your home	Two	Ref	
		Three	1.69 (0.06, 0.14)	0.44
		Four	3.15 (0.11, 0.21)	0.003
		More	40.09 (0.63, 0.76)	0.00
	How many people sleeps in the same room?	Only you or1	Ref	
		Two	3.15 (0.16, 0.28)	0.00
		Three	2.52 (0.13, 0.24)	0.01
		Four	2.27 (0.12, 0.22)	0.04
		more	6.47 (0.29, 0.43)	0.00
	What is your main lighting source?	Paraffin Lantern	Ref	
		Electricity	32.36 (0.74, 0.85)	0.00
		Others	0.80 (0.06, 0.14)	0.76
	What do you use to cook in your home?	Firewood	Ref	
		Charcoal	12.32 (0.67, 0.79)	0.00
		Gas	0.27 (0.03, 0.09)	0.00
		Electricity	0.17 (0.02, 0.07)	0.00
Knowledge of WASH (K-WASH)	Drinking contaminated water may cause diarrhea, stooling, stomach pain?	No	Ref	
		Yes	0.19 (3.39, 7.96)	0.15
	Drinking contaminated water may cause Cholera infection?	No	Ref	
		Yes	0.16 (4.07, 9.73)	0.18
	Cholera is a severe health problem which may cause death?	No	Ref	
		Yes	0.14 (4.69, 11.38)	0.21
	Open defecation may cause disease?	No	Ref	
		Yes	0.09 (7.02, 17.88)	0.29
	We should wash our hands before having food?	No	Ref	
		Yes	0.17 (3.89, 9.24)	0.18
Practices of WASH (P-WASH)	Is there a hand-washing station in your home?	No	Ref	
		Yes	27.56 (0.02, 0.06)	0.46
	Do you washed hands at all key times (before eating/cooking, after visiting toilet/cleaning babies)?	No	Ref	
		Yes	10.03 (0.06, 0.16)	0.27
	Do you used soap for hand-washing?	No	Ref	
		Yes	13.33 (0.05, 0.12)	0.32
	Is there pit latrine facility washing?	No	Ref	
		Yes	1.04 (0.65, 1.42)	0.00
	Is the pit latrine inside the house?	No	Ref	
		Yes	4.73 (0.14, 0.32)	0.14
	Is the pit latrine 10 m or half 10 m away from the house?	Yes	Ref	
		No	16.06 (0.59, 0.73)	0.00
		Not sure	2.35 (0.17, 0.29)	0.004
Waterborne-related disease	Have there been any incidence of outbreak of water borne disease in this village?	No	Ref	
		Yes	1045.44 (0.0003, 0.0030)	0.88
	Have there be any news of these related water borne disease outbreak/symptoms in this village?	Cholerae	Ref	
		Watery diarrhoea	2.73 (0.40, 0.54)	0.00
		Watery diarrhoea vomiting	0.36 (0.07, 0.15)	0.001
		Watery diarrhoea stomach pains	0.68 (0.13, 0.24)	0.34
	Do you know the source or curse of the outbreak?	No	Ref	
		Yes	13.57 (0.09, 0.11)	0.34
	Have there be any interventions?	No	Ref	
		Yes	12.57 (0.05, 0.13)	0.31
	What type of interventions?	PMTS	Ref	
		IH/CA	18.52 (0.17, 0.28)	0.00
		No response	213.77 (0.70, 0.82)	0.00

*GRW* ground running water, *PMTS* public modern toilet system, *IH/CA* improved healthcare/community awareness

*GRW* ground running water, *PMTS* public modern toilet system, *IH/CA* improved healthcare/community awareness.

The result presented in Table 3 shows that the reliability and consistency of the variables included in the PCA have a Cronbach’s alpha = 0.57. The Cronbach’s alpha rises to the highest of 0.92, acceptable consistency on dropping incidence of waterborne disease and intervention.

**Table 3:** Reliability to validate the consistency of the parameters included in PCA analysis

**Reliability if the item is dropped (with incidence and intervention)**						
**Variables**	**Raw *α***	**Std. *α***	**Cronbach’s *α* (95% CI)**	**Raw *α***	**Std. *α***	**Cronbach’s *α* (95% CI)**
**Water quality**	0.4	0.52	0.57 (0.38–0.62)	0.93	0.94	0.92 (0.91–0.94)
**Economic status**	0.29	0.44		0.87	0.9	
**Knowledge of WASH**	0.25	0.44		0.88	0.9	
**Practice of WASH**	0.33	0.5		0.91	0.93	
**Incidence of waterborne disease**	0.7	0.78		NA	NA	
**Intervention**	0.61	0.75		NA	NA	

### The linear relationship between knowledge and practice of WASH and selected study participants demographics using PCA

**Fig. 2:**
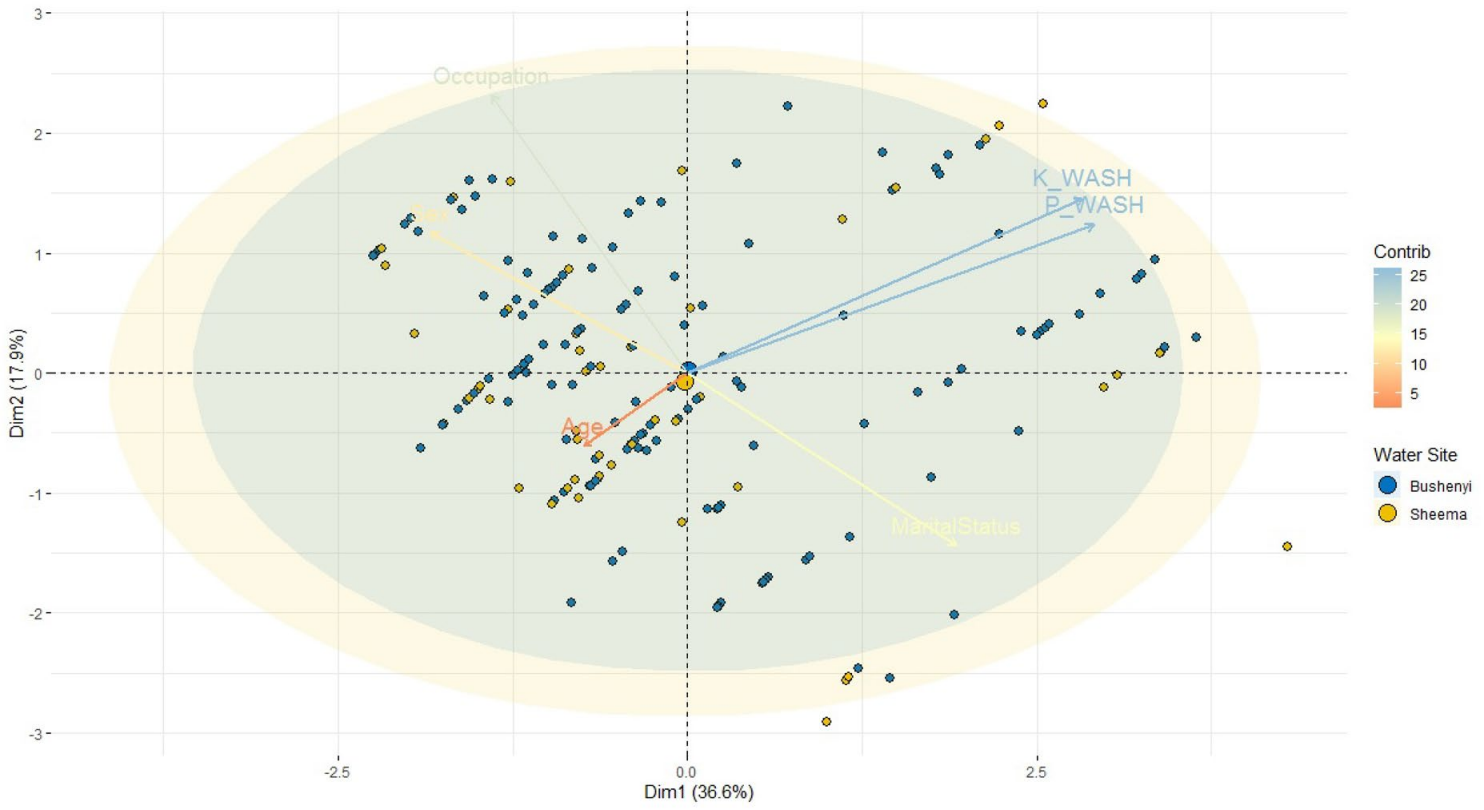
PCA graph of the linear relationship between knowledge and practice of WASH and selected study participants demographics

The principal component analysis (PCA) shows a linear relationship between knowledge and practice of WASH, with responses from selected respondent demographics as presented in Fig. 2 and S3 in supplementary material. The results indicate a linear relationship between the WASH attributes and the respondent responses on the state of water within the study area. In addition, the PCA 1 (referred to as Dim 1) accounts for 36.6% total variation which is a fair summary measure. However, it is imperative to note that knowledge and practice of WASH have a strong correlation, such that P_WASH (*r* = 0.84, *p* < 0.001), K_WASH (*r* = 0.82, *p* < 0.001), and marital status (*r* = 0.55, *p* < 0.001) were all positively associated with PCA 1, whereas age (*r* = −0.21, *p* < 0.001), occupation (*r* = −0.40, *p* < 0.001), and sex (*r* = −0.53, *p* < 0.001) were negatively associated. Also, a similar association was observed by correlation coefficients in the result shown in Tables 4 and 5. The PCA 2 (Dim 2) explains the total variation of 17.9% such that occupation (*r* = 0.67 *p* < 0.001), K_WASH (*r* = 0.425, *p* < 0.001), P_WASH (*r* = 0.35, *p* < 0.001), and sex (*r* = 0.33, *p* < 0.001) have a strongly positive association, whereas age (*r* = −0.17, *p* < 0.001) and marital status (*r* = −0.41, *p* < 0.001) were negatively associated. The PCA 3 (Dim 3) reveals 15.3% variation which is influenced by age, sex, and economic status. The PCA 4, 5, and 6 show 9.7%, 4.5%, and 3.8% variation respectively. Based on the two distinct levels of the knowledge of WASH, i.e., good/bad (> 50 and < 50% scores), 71% and 29% of the study participants score high and low. On the practice of WASH, i.e., proper/improper score, 32% and 68% of the study participants score high and low respectively.

**Table 4:** Pearson’s correlation analysis of knowledge and practice of WASH, water quality, basic economic status, and waterborne disease

		**K_WASH**			**P_WASH**			
		***r* (95% CI)**		***p*-value**	***r* (95% CI)**		***p*-value**	
**High knowledge category (> 50%)** **P_WASH** **K_WASH**		0.72 (0.63 to 0.78) 1		< 0.001 -	1 0.75 (0.68 to 0.80)		- < 0.001	
**Low knowledge category (< 50%)** **P_WASH** **K_WASH**		−0.39 (0.34 to 0.78) 1		0.002 -	1 0.40 (0.27 to 0.52)		- < 0.001	

	**WQ**		**ECO_STAT**		**QI9CD**		**Qwbd6CD**	
	***r* (95% CI)**	***p*-value**	***r* (95% CI)**	***p*-value**	***r* (95% CI)**	***p*-value**	***r* (95% CI)**	***p*-value**
**WQ**	1	-	0.66 (0.57–0.73)	*p* < 0.001	−0.25 (−0.37 to −0.12)	*p* < 0.001	−0.23 (−0.35 to −0.09)	*p* = 0.001
**ECO_STAT**	0.66 (0.57–0.73)	*p* < 0.001	1	-	−0.25 (−0.37 to −0.12)	*p* < 0.001	−0.22 (−0.36 to −0.09)	*p* = 0.001
**QI9CD**	−0.25 (−0.37 to −0.12)	*p* < 0.001	−0.11 (−0.24 to 0.03)	*p* = 0.133	1	-		
**Qwbd6CD**	−0.23 (−0.35 to −0.09)	*p* = 0.001	−0.22 (−0.36 to −0.09)	*p* = 0.001	-	-	1	-

*r* Pearson’s correlation coefficient, *QI9CD* incidence of waterborne disease, *Qwbd6CD* waterborne-related disease

**Fig. 3:**
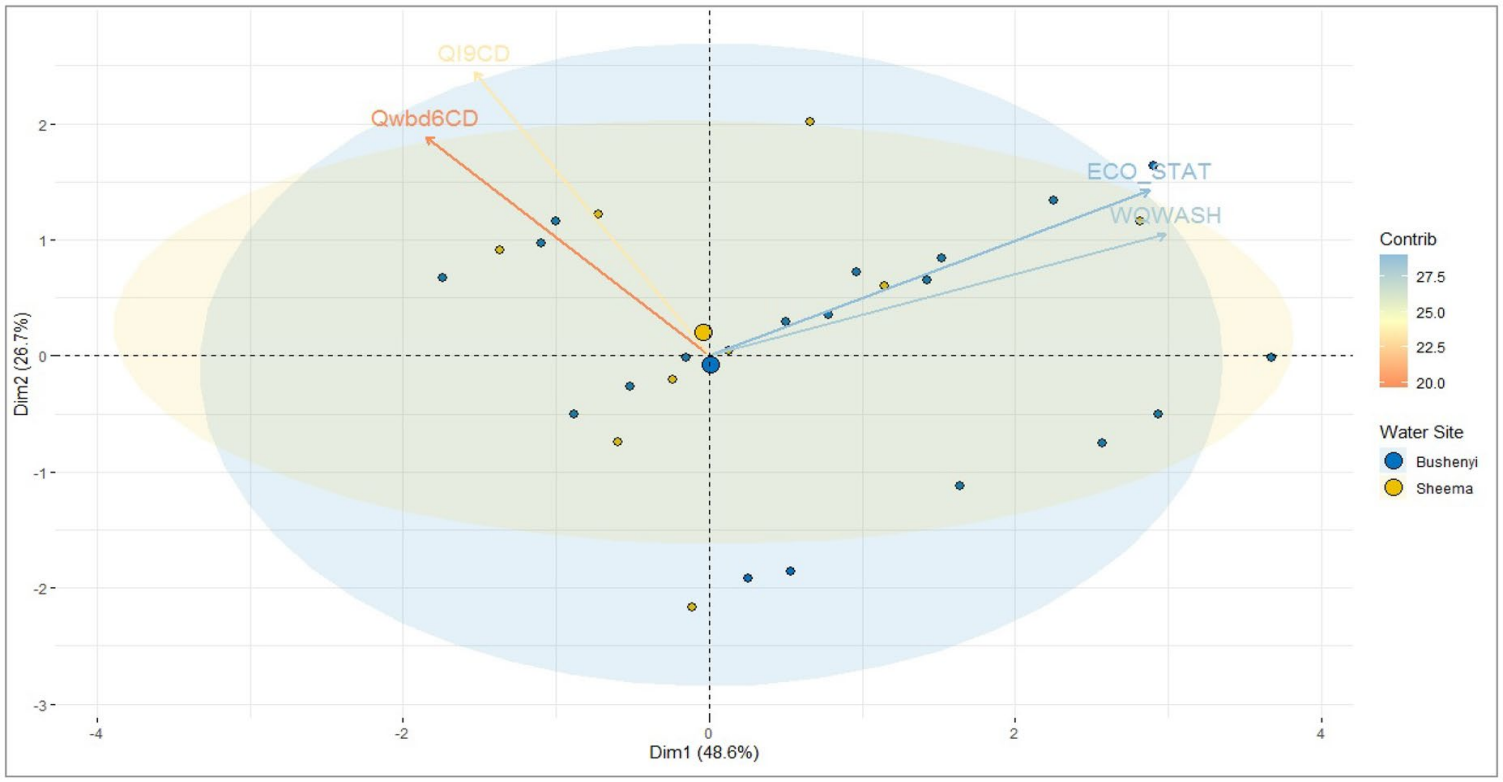
PCA graph of the linear relationship between water quality and economic status and intervention and incidence of WBD. QI9CD, questions on intervention coded; Qwbd6CD, questions on waterborne disease coded

Similarly, we used the principal component analysis (PCA) to understand the variation and relationship between water quality, economic status, disease incidence, and interventions as presented in Fig. 3 and S3.

The results indicate a strong positive linear relationship between the water quality and economic status; also, a constantly strong liner relationship exists with incidence of disease and interventions. However, the attributes in the PCA 1 (Dim 1) account for a total variation of 48.6% given an average summary measure. These observations note a positive association of water quality (WQWASH) (*r* = 0.86, *p* < 0.001), economic status (ECO_STAT) (*r* = 0.83, *p* < 0.001), and incidence of waterborne disease (Qwbd6CD) (*r* = −0.53, *p* < 0.001); interventions (QI9CD) (*r* = −0.44, *p* < 0.001) are negatively associated with PCA 1. The PCA (Dim 2) explains total variation of 26.7% such that QI9CD (*r* = 0.71, *p* < 0.001), Qwbd6CD (*r* = 0.54, *p* < 0.001), ECO_STAT (*r* = 0.41, *p* < 0.001), and WQWASH (*r* = 0.30, *p* < 0.001) variables are all positively associated. The PCA (Dim 3) depicts a 15.3% total variation which most likely has been influenced by economic status. The PCA 4, 5, and 6 show 9.7%, 4.5%, and 3.8% variation respectively with less impact (Fig. 3). For water quality, i.e., safe and/or unsafe, 36% and 64% of the study participants score high and low, while for the basic economic status, i.e., stable and/or unstable, 43% and 57% of the study participants score high and low.

**Fig. 4:**
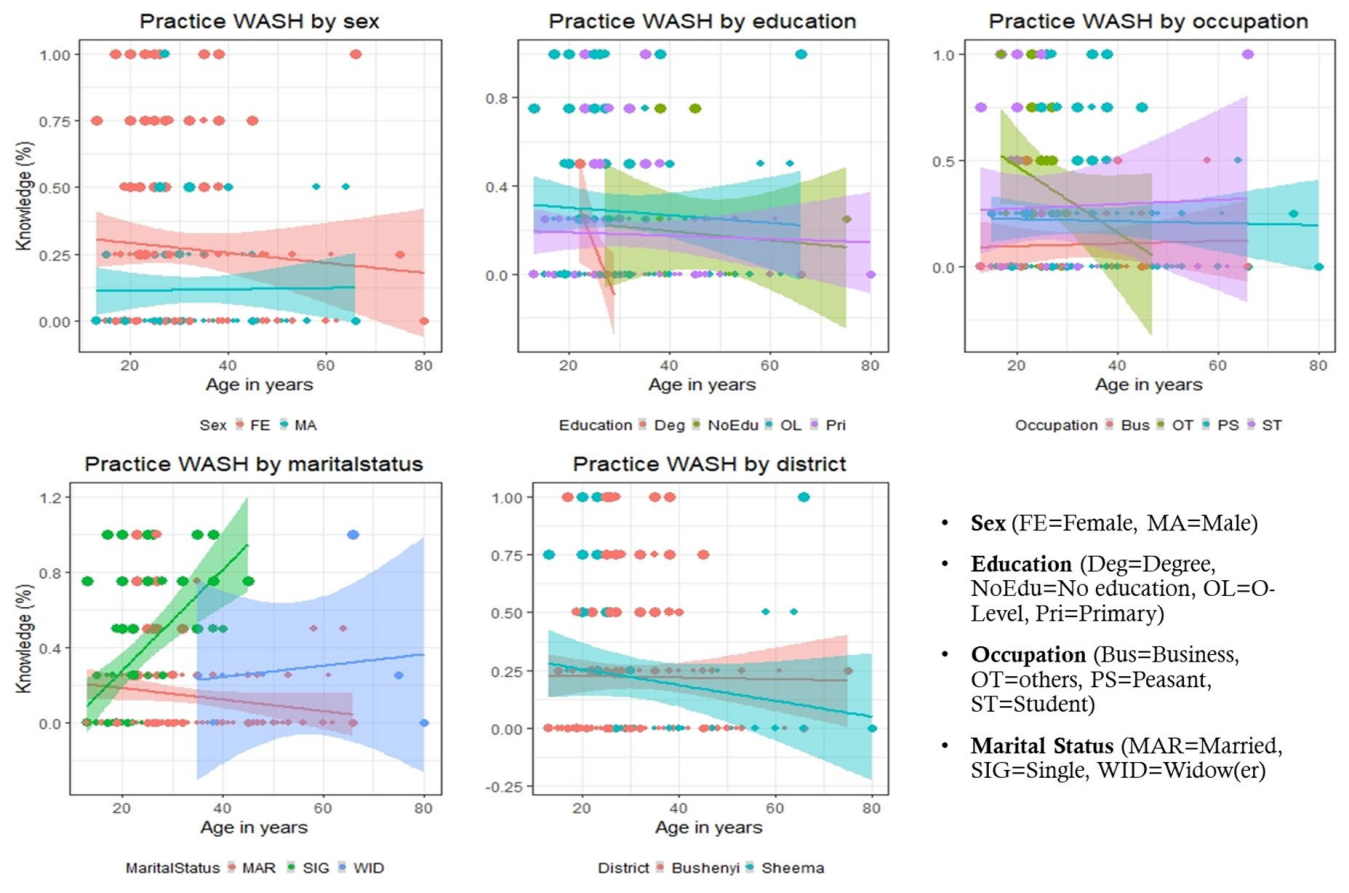
Analysis of linear regression of study participants demographic characteristics and knowledge and practice of WASH

The findings as shown in Table 4 and Fig. 4 reveal that there is a linear relationship between knowledge of WASH and practice of WASH while showing an opposite relationship with age, sex, and occupation and a positive relationship with marital status as also shown in the PCA (Dim 1) (Fig. 2). A similar observation is recorded when considering the association between basic economic status and water quality. Dim 2 on occupation has shown its primary contributing attribute as it has influenced the positive end. The result obtained for the PCA on water quality indicates that basic economic status is highly correlated with water quality, while waterborne disease and interventions are negatively contributing to Dim 1.

### Study participants demographic characteristics and knowledge and practice of WASH

Description of demographic characteristics by the age of the study participants responses and the linear variation of knowledge and practice of WASH are depicted in Fig. 4. The corresponding-coloured lines indicate the linear regression line of the demographic characteristic, while the size of the points reflects the score of knowledge and practice of WASH, respectively. These scores ranged between 0.0 and 1.0 and 8 and 80 on the knowledge and practice scales respectively. However, the female has more knowledge than the male; as age increases, the practices of WASH decrease as female age increases, and the male has a common knowledge but is stable as age increases. Those with a degree have a better knowledge of WASH. However, a slight increase in age declines with the practice of WASH. The business group knows WASH excitedly from the occupation perspective but declines with age increases.

On the other hand, married has better knowledge with an increase in age. However, single has common knowledge of WASH with a spontaneous increase in practices of WASH. Sheema districts have a slightly better knowledge of WASH with a decrease in practice as age increases. At the same time, Bushenyi has a steady practice of WASH with an increase in age.

### The analysis of associations between knowledge and practices of WASH, water quality, economic status, and waterborne-related disease

**Table 5:** Logistic regression analysis of knowledge, practice of WASH, and selected study participants demographics

**Category**	**Questions**	**Response**	***OR*** **(2.5 to 97.5% CI)**	***p*** **-value**	**Category**	**Questions**	**Response**	***OR*** **(2.5 to 97.5% CI)**	***p*** **-value**
Demographic/K_WASH	Sex	Female	Ref		Demographic/P_WASH	Sex	Female	Ref	
		Male	0.68 (0.35, 1.31)	0.15			Male	0.72 (2.45, 1.15)	0.178
	Age	Under 18	Ref			Age	Under 18	Ref	
		19–30 years	2.53 (0.98, 7.05)	0.065			19–30 years	2.92 (1.42, 6.25)	0.005
		31–42 years	6.34 (2.15, 20.26)	0.026			31–42 years	3.99 (1.76,9.39)	0.001
		43 and above	3.54 (0.97, 13.27)	0.057			43 and above	2.85 (1.10, 7.48)	0.032
	Education	Degree	Ref			Education	Degree	Ref	
		O level	9.53 (1.49, 165.57)	0.047			O level	2.45 (0.74, 9.99)	0.170
		Primary	4.55 (0.72, 79.06)	0.172			Primary	2.32 (0.71, 9.34)	0.193
		No education	3.66 (0.42, 73.22)	0.292			No education	2.52 (0.59, 12.19)	0.225
	Occupation	Business	Ref			Occupation	Business	Ref	
		Others	6.86 (2.58, 19.58)	< 0.001			Others	5.07 (2.48, 10.67)	< 0.001
		Peasant	1.68 (0.75, 4.05)	0.223			Peasant	1.46 (0.81, 2.76)	0.221
		Student	0.92 (0.34, 2.59)	0.274			Student	1.95 (0.91, 4.27)	0.089
	Marital Status	Married	Ref			Marital Status	Married	Ref	
		Single	6.95 (3.59, 13.93)	< 0.001			Single	4.58 (2.82, 7.55)	< 0.001
		Widow	2.05 (0.48, 7.59)	0.3			Widow	1.94 (0.67, 5.17)	0.198
	Districts	Bushenyi	Ref			Districts	Bushenyi	Ref	
		Sheema	0.96 (0.53, 1.72)	0.89			Sheema	1.13 (0.71, 1.78)	0.592
K_WASH	Drinking contaminated water may cause diarrhea, stooling, stomach pain?	No	Ref		P_WASH	Is there pit latrine facility at your home?	No	Ref	
		Yes	6.31 (0.04, 9.26)	< 0.001			Yes	0.12 (2.45, 5.14)	< 0.001
	Drinking contaminated water may cause cholera infection?	No	Ref			Is the pit latrine 10 m or half 10 m away from the house?	Yes	Ref	
		Yes	5.85 (0.38, 8.83)	0.05			No	0.25 (0.16, 0.41)	< 0.001
	We should wash our hands before having food?	No	Ref				Not sure	0.44 (0.28, 0.69)	< 0.001
		Yes	4.07 (0.27, 5.95)	< 0.001					

Ref variables: sex (female), education (degree), occupation (business), marital status (married), and district (Bushenyi)

### Knowledge and practice of WASH, water quality and basic economic status, and incidence of waterborne disease

The relationship determined by Pearson’s correlation analysis is shown in Table 4. The result by knowledge of WASH dichotomy depicts knowledge has a strong positive correlation with practice of WASH (correlation coefficient = 0.72; 0.75; and *p*-values < 0.001; < 0.001 respectively). On the other hand, water quality is positively significantly associated with basic economic status and inversely correlated with waterborne disease incidence (0.66; −0.25; −0.23 and *p* < 0.001, *p* < 0.001, *p* = 0.001). Incidence of waterborne disease has negative correlation coefficients of −0.23 and −0.22 and *p-value* of 0.001.

The multivariable logistic regression model was used to examine the statistically significant association of the demographics with knowledge and practice of WASH in Table 5 and water quality, basic economic status, and waterborne-related disease in Table 6. The result indicates that age has no significant linear relationship with knowledge of WASH. Sex is not significant with knowledge of WASH, although the male has a better knowledge of WASH. Population with a degree has better knowledge of WASH, but it is not significantly associated (*p* > 0.05). Occupation is significantly associated with WASH knowledge such that business has about six times more knowledge (*p* = 0.001, *OR* = 6.86) than other occupations (technician, hair-dressers, bike riders), while peasant and student have no difference. Marital status was significantly associated with WASH knowledge the married have more knowledge (*p* < 0.001, *OR* = 6.95) than single, while the widow(er) has no difference. Respondents that answered yes to “drinking contaminated water may cause diarrhea, stooling, stomach pain; may cause cholera infection; and we should wash our hands before having food” have a high score on WASH knowledge. Similarly, age, sex, education, and districts have no significant relationship with WASH practice. Sex is not significant with WASH practice, although the male has a better practice of WASH. Population with a degree has a better practice of WASH, but it is not significantly associated (*p* > 0.05). Occupation is significantly associated with WASH practice such that business exercises better practice (*p* < 0.001, *OR* = 5.07) compared to other occupations (technician, hair-dressers, bike riders), while peasant and student have no difference. Marital status was significantly associated with WASH practice such that married exercises better practice (*p* < 0.001, *OR* = 4.58) compared to single, while widow(er) has no difference in Table 6. Respondents who answered that they have pit latrine facility at their homes are likely to have a low score on WASH practice. With regard to water quality, the model shows the sources of water use to association knowledge of WASH, such that the population that uses tap/borehole water is likely to have a more knowledge of WASH (Table 6).

**Table 6:** Logistics regression analysis of the water quality, basic economic status, and waterborne-related disease

**Category**	**Questions**	**Response**	***OR*** **(2.5 to 97.5% CI)**	***p*-value**
WQ	Which of these sources of water do you use?	Open spring	Ref	
		Ponds/GRW	0.34 (0.22, 0.52)	0.012
		Lake	0.46 (0.34, 0.63)	0.001
		Tap/borehole water	0.45 (0.31, 0.64)	< 0.001
Basic economic status	Do you own a house, land?	No	Ref	
		Yes	0.42 (0.04, 1.41)	0.15
	Do you own radio, television?	No	Ref	
		Yes	0.40 (0.15, 1.01)	0.06
	How many people sleeps in the same room?	Two	Ref	
		Three	0.89 (0.21, 4.01)	0.881
		Four	0.74 (0.16, 3.40)	0.690
		More	0.68 (0.19, 2.59)	0.553
	What do you use to cook in your home?	Firewood	Ref	
		Charcoal	2.89 (0.82, 14.75)	0.141
		Gas	1.48 (0.04, 16.99)	0.772
		Electricity	3.47 (0.06, 51.05)	0.411
Eco_Stat and WBD	Have there be any incidence of outbreak of water borne disease in this village?	No	Ref	
		Yes	0.84 (0.049, 1.48)	0.541
WQ_WASH and WBD	Have there any incidence of outbreak of water borne disease in this village?	No	Ref	
		Yes	0.66 (0.50, 0.88)	0.005

*WBD* waterborne-related disease

### Thematic qualitative analysis of study participants responses on WASH among the study population

#### Sources of water versus water quality

Among the theme that arose during the investigation and study participants face-to-face interviews was water source as a significant influencing determinant of water quality. The sampled population of the study does not have access to potable water supply; therefore, the alternative and the most frequent water source was surface water such as a lake, wells, running water (RW), and open springs. Most of their responses negate the study, stating that fetching and storage may expose their water to contamination, contributing to poor water quality. As a result of their negative view of the above, the population uses a Matooke finger to cover 20 L of jerrycans of water for storage compared to the possible regulatory standard water storage method in covered jerrycans. The majority of the study participants consistently use the statement below:

Since I was born, I have never seen my mother and others use any cover on jerrycan, but they have always taken one matooke finger to cover jerrycan, and they are safe; hence I will also use it. I do not have to look for new coverage to buy (study participant Igara East Bushenyi District).

The above observation indicates unsafe routine practice among some group of the population study. This practice is a potential risk hotspot to water contamination at the collection point or fetching and storage.

#### Study participants versus sanitation and hygiene

One of the influencing themes raised by study participants’ face-to-face interviews on sanitation and hygiene is the location of a pit latrine and open defecation. About 60% of the face-to-face responses were, “I fear to go to pit latrine located 10 m or 6 m away from the house; I cannot manage”. While the peasant farmers argue that open defecation is normal as it contributes to soil fertility, there is no need for a toilet system. However, this will have contributed negatively to the improper practices of WASH.

#### Basic economic status influencing factors to water quality and incidence of disease

One of the critical variables that are kin to the incidence of waterborne disease is the basic economic status. For instance, during the interview session, some informants argued that the primary reason for not boiling water was the lack of electricity (umeme) in the locality.

There is no umeme in this village; how do I boil water with firewood wait to cold before drink. I cannot manage. I have been drinking this lake water since I was born without any treatment. I am not sick even when I am sick; I take herbs (study participant at Kantungu lake).

## Discussion

The implementation of WASH is critical among most of the population living in hard-to-reach settlements in low-income countries. Therefore, this study generated data from distilling socio-anthropological to identify demographic characteristics linked to WASH variables by combining quantitative and qualitative data in WASH investigations. Our study is in line with the components presented in the hygiene improvement framework report 2004 (EHP/UNICEF/WES/USAID, 2004), which includes improving access to water and sanitation, also called “Hardware”, promoting hygiene and strengthening the enabling environment. Despite the high score on the knowledge of WASH, the practice of WASH and water sources score is relatively low score observation from the study participants response. Our study indicates unimproved water sources/quality among the study population, which implies unsafe water while 78% of the population do not treat their water and very few boil their water before drinking and other uses. Our findings are in agreement with the previous reports that improved water sources are classified as self-supply water from shallow wells (Nayebare et al., 2020) and deep boreholes (Walekhwa et al., 2022), and none of the sources was classified as safe, open/unprotected springs that could be potentially contaminated with pathogenic bacterial (Gebremichael et al., 2021; Hotez et al., 2006). In low-income countries, the high burden of diarrhoea is linked to poor access to safe and sufficient water sources (WHO/UNICEF, 2012, 2015) and poor sanitation and hygiene. This study findings are comparable to investigations conducted in Kampala and Nsazi Island Uganda, Lilongwe Malawi, northwest Ethiopia, Siaya County, Kenya, Angola, Ghana’s Tamale Metropolitan Area, South African villages, and Nigeria (Azage et al., 2016; Boakye-Ansah et al., 2016; Buckley & Kallergis, 2019; Kapwata et al., 2018; Nakagiri et al., 2015; Nygren et al., 2016; Yaya et al., 2018). WASH conditions in hard-to-reach communities of low-income countries are frequently unsatisfactory due to low budgets, a lack of capability, unclear legislation, and a lack of realistic options to offer services by all arms of government (Andersson et al., 2016; WHO/UNICEF, 2015; WHO et al., 2016; Bain et al., 2018; Mara & Evans, 2018). However, self-operational portable water and sanitation facilities are challenging to maintain among a population living in poor resource settings, thereby facilitating the transmission of diarrhoeagenic pathogens and related diseases (Igere et al., 2022; Nayebare et al., 2020; O’Keefe et al., 2015; Onohuean et al., 2022a, b). WASH can thwart the global disease burden by 9.1% and decrease the death rate by 6.3%. In addition, improved water quality or sources can curb 21% of diarrhoea morbidity, as adequate sanitation diminishes diarrhoea morbidity by 37.5% (Clasen et al., 2015). On the other hand, simple practices of washing hands at every critical time can decrease the number of diarrhoea cases by 35%. Interestingly, 45% of diarrhoea episode reduction can be achieved by enhancing drinking-water quality combined with point-of-use disinfection (Annika Launiala, 2009).

### The linear relationship between knowledge, practice of WASH, and selected study participants demographics

The assumption of the linear relationship between knowledge and practice is akin to awareness campaigns and implementation in public health intervention strategies (Annika Launiala, 2009; Muleme et al., 2017). The result obtained from our PCA indicates a strong positive linear relationship between knowledge and practice of WASH, which is implicated in promoting awareness implemented and public health interventions. This relationship points to the fact that adequate knowledge and effective WASH practice could be a positive solution to curbing the spread of water-related diseases and outbreaks. This agrees with Bartram and Cairncross’s (2010) report that washing hands with soap, drinking treated water, and proper disposal of excreta have reduced the risk and incidence of diarrhoea from 48 to 17%. However, this is a rarely observed linear relationship (Mosca et al., 2000). Our findings, therefore, validate and support the awareness strategy as a critical target for the implementation of the public health intervention system (Warwick, 1983; Annika Launiala, 2009).

Notwithstanding, the result also implies that the relationship does not apply to all the study population. Furthermore, to ensure that our analysis retains context, selected study participants demographics were maintained (age, sex, occupation, education level, marital status), resulting in internal consistency and reliability of Cronbach’s alpha = 0.50. Several studies have revealed that demographic factors variably influence WASH. Our finding implies that occupation and marital status significantly affects the knowledge and practice of WASH; this is similar to other studies that age (Lewoyehu, 2021; Morgenroth, 2014), gender (Koskei et al., 2013), occupation (Koskei et al., 2013), and education level (Ashaolu & Onundi, 2014) are associated with an adequate supply of safe drinking water and other WASH conditions. However, the selected demographics attributes offer no categorical context to the WASH measurement but influence the success of WASH.

### The linear relationship between water quality, basic economic status, incidence of waterborne diseases, and interventions

The findings from the PCA of the attributed variables show a linear relationship between individual variables such that water quality is linearly related to basic economic status. Similarly, the incidence of waterborne disease is linearly related to interventions. This implies that a “stable” basic economic status has a concomitant influence on “good” water quality. Same in the revise, the “unstable” basic economic status of a population tends to have a concomitant influence on “bad” water quality. Stable and unstable basic economic status significantly impacts disease outbreaks’ incidence and interventions. This conforms with the study conducted by various investigators (Clasen, 2015; Fewtrell et al., 2005), suggesting that water treatment alone at home can significantly reduce death due to diarrhoeal, even without the combination of other additional measures. Our study reveals that diarrhoea is a common health issue among the studied population with few intervention programs, indicating a brewing risk of waterborne disease outbreak potential in the future. However, improving water quality or rapid water quality assessment and improved chlorination is a keen intervention for preventing diarrhoea or response to acute diarrhoea (Clasen et al., 2015; Rajasingham et al., 2020). Although we obtain Cronbach alphas of 0.57 due to the low responses to question on incidence and intervention, it is important to note that a high Cronbach’s alpha = 0.92 for internal consistency and reliability was obtained by maintaining all categorical context measurements in the PCA.

### Factors affecting WASH variable axiom

The logistic regression and the qualitative thematic analysis were used to explain a linear relationship between the WASH variable and study participants demographics. Results obtained from this model indicate that occupation and marital status is statistically significant and associated with knowledge and practice of WASH. This is similar to the findings of Koskei et al. (2013), Baye et al. (2012), Geremew Gebremichael et al. (2020), and Gualie and Enyew (2019) that occupation significantly influences household water sources and/or uses and marital status of the household head has a substantial impact on WASH, specifically type of toilet facility used by families (Koskei et al., 2013). Surprisingly, individuals who engage in other occupations (manual labourers, bike riders, technicians, male and female hair-dressers) were ~ 7 and ~ 5 times better with awareness and practices of WASH, respectively, than commercial occupation (traders, small business). However, the reasons behind such disparity are not very clear from the interview and investigative discussion. Nevertheless, an impoverished population carries the most significant burden of unsafe water quality, poor sanitation, and hygiene. It is possible they are not ignorant of WASH, but the unstable primary economic status has left them with no alternative.

### Implication for public health

Most diarrhoeagenic infections/diseases are endemic in the area associated with poor implementation of WASH, whereas others are epidemic in nature, especially cholera and typhoid fever. However, the WASH facility is an effective intervention within emergency sceneries and longer-term development (Brown et al., 2012, 2015) but emergencies often present more challenging situations for WASH implementation. Sympathetically, Ramesh et al.’s (2015) systematic review reports that for the past 33 years, only six studies have assessed WASH intervention concerning public health outcomes and evaluated water-related interventions, with just one study on hygiene, but none of the studies provided evidence on the impact of sanitation interventions. Our study has empirically proved the linear relationship between knowledge and practice of WASH by using data collected from populations usage of surface waters in two districts of western Uganda. Besides, we show that the water quality and basic economic status determine whether this linear relationship grips or not to inform public health interventions and strategies on water-related diseases and outbreak. Also, our findings suggest that adequate knowledge and improved sanitation implementation might result in a desirable change in the spread and incidence of waterborne diseases. Therefore, improved WASH services and practices at personal practicing and supporting hygiene behaviours may have a positive impact at the community level, also ensuing the creation of hand washing and drinking water stations and the training of village health teams (VHT) on hand hygiene awareness and campaigns, in rural communities. The user-friendly analytics from our dataset will guide WASH researchers and allow a practicable R-code to users for testing the WASH linear relationship and evaluation of public health intervention.

### Study limitations

The major limitation of this study is the determination of sample size. However, we acknowledge that the recruited respondents may have compromised, thereby influencing the outcome of our study. Also, this study included a population of remote villages, which may have impacted the level of knowledge and practice of WASH as we reported (due to education levels, economic status, and awareness of health information). Lastly, we limit our inference to the participant that settles within 200 m circumference of the surface waters in the two districts as the population may not be a good representative of the general population.

## Conclusion

The results reveal uniformities and discrepancies per the WASH linear relationship and identify associated demographic factors. Despite the knowledge of WASH, the basic economic status highlights why “low economic population groups” in remote settings may not effectively practice WASH. The diarrhoea was common among low basic economic status in the study population. Our findings suggest a need for advocacy for WASH and a firm practice of WASH to effectively subside the common diarrhoea and prevent potential waterborne disease outbreaks. It will be necessary for every household to treat, boil, and chlorinate drink water and have a washing station.

## Supplementary Information

Below is the link to the electronic supplementary material.

Supplementary file1 (DOCX 26 KB)

Supplementary file2 (DOCX 94 KB)

Supplementary file3 (DOCX 19 KB)

## Data Availability

The datasets/information used for this study are available within the manuscript and the supplementary materials.

## Abbreviations

WASH: Water quality, sanitation, and hygiene
SDG 6: Sustainable Development Goal 6
WHO: World Health Organization
UNICEF: United Nations International Children’s Emergency Fund
NTDs: Neglected tropical diseases
KIU: Kampala International University
LC1: Local government chairperson
PCA: Principal component analysis
CIs: Confidence intervals
