# Supplementary material for: Demographic dynamics of waterborne disease and perceived associated WASH factors in Bushenyi and Sheema districts of South-Western Uganda
Source: Environ Monit Assess. 2023 Jun 20;195(7):864. doi: 10.1007/s10661-023-11270-1 (PMC10281895; doi:10.1007/s10661-023-11270-1)
Supplement: Supplementary file 1 — Supplementary file1 (DOCX 26 KB) [file 10661_2023_11270_MOESM1_ESM.docx]

**Survey Questionnaire**

Principal Investigator: Hope ONOHUEAN

Study Title: Demographic dynamics of water-borne disease and associated WASH factors in Bushenyi and Shema districts of South Western Uganda

Ethical Approval: The study was review and approved by the ethics committee of Kampala International University (KIU), Western campus, Ishaka-Bushenyi Uganda

(UG-REC-023/201919).

Introduction: Thank you for agreeing to speak with me today. I am going to ask you questions about Water quality, sanitation and hygiene (WASH) and associated water-borne disease. Please feel free to let me know if you are not comfortable with answering any questions and I will move on to the next question.

| **Section 1. Personal History and Demographics** | | | |
| --- | --- | --- | --- |
| **No.** | **Item Wording** | **Response Options** | **SKIP** |
| 101 | How old are you? | _____________ [YEARS] |  |
| 102 | What is your highest level of education completed? | (Degree)Higher education=0  (S5-S6) A-level=1  (S1-S4) O-level=2  (P1-P7) Primary=3  No Education /Never went to school =4  No response =5 |  |
| 103 | What is your Marital status? | Married=0  Single=1  Divorce=2  Widow=3  Widower=4  No response=5 |  |
| 104 | What is your Occupation? | Student=0  Peasant Farmer=1 Fisherman =2  Business=3  Health worker=4  Others =5 |  |
| 105 | What is your ethnic group? | Banyankole=0  Basoge=1  Musoga=2  Muryanguru=3  Mukiga=4  DRC=5  Munyoro=6  Munganwanda=7  Others=8  No response=9 |  |
| 106 | The study site or areas? | Kancece=0  Katuto B=1 Bugaramma=2  Katunga Lake=3 Nyarutuntu=4  Ishaka=5  Kitagata=6  Orushenyi spring=7 Bugaya=8  Kibasi=9  Kirera1=10  Kayembe=11  Rutoto=12 Nyakashare=13 Rwongoma=14 Byegyane=15 Karambi=16  Rijumo=17  Nyabure cell Omukagaye=18 Omukayeole Rakandu=19, Omukayenbe=20, Omukashesho Nyarmwaroke=21  Kagogo=22 Nyamweranole11=23 Kagogo Nyamwerande=24  Omukasoga=25  Bunera central=26 |  |
| 107 | Which Districts /Villages is this? | Sheema/Kabwohe=0  Bushenyi/Nyamizinga Bushenyi=1  Bushenyi/Rwamuro Nyabukurunga=2  Bushenyi/Katungu lake=3  Bushenyi/Katungu Tap=4  Bushenyi/Katungu spring=5  Sheema/Kitagata=6  Bushenyi/Orushenyi Ishaka spring=7 |  |
| 109 | What is your religion/faith based? | Christian=0  Muslim=1  Others=2 |  |
|  | **Section 2. Water Sources** |  |  |
| 201 | What is the main source of water for the village? | Spring/well=0  Lake=1  River=2  Tap Water=3  Ground running water=4  Others=5 |  |
| 202 | Which of these sources of water do you use? | Spring/well=0  Lake=1  River=2  Tap Water=3  Ground running water=4  Others=5  Spring/well+Lake=6, Spring/well+Tap Water=7  Lake+ Tap Water=8  Spring/well+ Lake+ Tap Water=9  Tap Water+ Ground running water=10  Spring/well+ Tap Water+ Ground running water=11  Spring/well+ Ground running water=12 |  |
| 203 | How do you fetch the water? | Bucket =0  Cup =1  Bottles=2  Jerrycan=3  Others=4  Bucket+ Jerrycan=5 |  |
| 204 | Do you treat your water? | Yes=0  No=1 |  |
| 205 | If yes what type of water treatment is common in the village? | Boiling=0  Chlorination=1  Filtration=2  Sedimentation=3  Others=4 |  |
| 206 | How do you store/keep your house use water? | Water pot=0  Drum=1  Jerrycan=2  Tank=3  Others=4  Drum + Jerrycan=5 |  |
|  | **Section 3. Basic Economic Status** |  |  |
| 301 | What is your current income? | ____ /day/week/month? |  |
| 302 | Do you own a house, Land? | Yes= 0  No= 1 |  |
| 303 | Do you own radio, television? | Yes= 0  No= 1 |  |
| 304 | How many people stay in your home | Only me or 1=0  2=1  3=2  4=3  More=5 |  |
| 305 | How many people sleeps in the same room? | Only me or 1=0  2=1  3=2  4=3  More=5 |  |
| 306 | What is your main lighting source? | Paraffin lantern=0  Electricity=1  Others=3 |  |
| 307 | what do you use to cook in your home? | Firewood=0  Charcoal=1  Gas=2  Electricity=3  Others=3  Paraffin=4 |  |
|  | **Section 4. Knowledge on Hygiene & Sanitation** |  |  |
| 401 | Drinking contaminated water may cause diarrhea, stooling, stomach pain? | Agreed=0  Strongly agreed=1  Disagreed=2  Strongly Disagreed=3  Others=4 |  |
| 402 | Drinking contaminated water may cause Cholera infection? | Agreed=0  Strongly agreed=1  Disagreed=2  Strongly Disagreed=3  Others=4 |  |
| 402 | Cholera is a severe health problem which may cause death? | Agreed=0  Strongly agreed=1  Disagreed=2  Strongly Disagreed=3  Others=4 |  |
| 403 | Open defecation may cause disease? | Agreed=0  Strongly agreed=1  Disagreed=2  Strongly Disagreed=3  Others=4 |  |
| 404 | We should wash our hands before having food? | Agreed=0  Strongly agreed=1  Disagreed=2  Strongly Disagreed=3  Others=4 |  |
|  | **Section 5. Practices of Hygiene & Sanitation** |  |  |
| 501 | Is there a hand-washing station in your home? | Yes=0  No=1 |  |
| 502 | Do you washed hands at all key times (before eating/cooking, after visiting toilet/cleaning babies)? | Yes= 0  No= 1 |  |
| 503 | Do you used soap for hand-washing? | Yes=0  No=1 |  |
| 504 | Is there pit latrine facility washing? | Yes =0  No=1 |  |
| 506 | Is the pit latrine inside the house? | Yes=0  No= 1 |  |
| 507 | Is the pit latrine 10 meters or half 10 meters away from the house? | Yes=0  No=1  Not sure=2 |  |
| 508 | If no, could you tell us, why is not half 10 meters away from the house? | ----------------- |  |
|  | **Section 6. water borne disease** |  |  |
| 601 | Have there be any news of these related water borne disease symptoms in this village? | Cholerae=0  Watery diarrhea=1  Vomiting=2  Stomach pains=3  Others=4  Watery diarrhea+ vomiting=5  watery diarrhea+ Stomach pains=6  Vomiting+ Stomach pains=7 |  |
| 602 | Have there be any news of outbreak of water borne disease in this village? | Yes=0  No=1 |  |
| 603 | Do you know the source or curse of the outbreak? | Yes=0  No=1 |  |
| 604 | If yes, can you mention it / them | ----------------- |  |
|  | **Section 7. Recommendation** |  |  |
| 701 | Is there any other useful information not captured above? | Yes=0  No=1 |  |
| 702 | Do you have any suggestion for the prevention of water borne disease? | Yes=0  No=1 |  |
| 703 | If yes, what are those suggestions? | ---------------------- |  |

Thank you for your participation.
