# Supplementary material for: Demographic dynamics of waterborne disease and perceived associated WASH factors in Bushenyi and Sheema districts of South-Western Uganda
Source: Environ Monit Assess. 2023 Jun 20;195(7):864. doi: 10.1007/s10661-023-11270-1 (PMC10281895; doi:10.1007/s10661-023-11270-1)
Supplement: Supplementary file 2 — Supplementary file2 (DOCX 94 KB) [file 10661_2023_11270_MOESM2_ESM.docx]

**(S 2 Text) consent Letter**

**Title:** **Demographic dynamics of water-borne disease and associated WASH factors in Bushenyi and Shema districts of South Western Uganda**

**Principal Investigator: Hope ONOHUEAN**

**Address: School of Pharmacy, Dept. Pharmacology &Toxicology, KIU Western Campus, Ishaka-Bushenyi, Uganda.**

**Research General Information.**

This is for only academic research purpose. This study is to investigate water borne related diseases, diarrhoea and cholera infection/outbreak. I am only collecting information necessary to better understand the diarrhoea and cholera infection/outbreak associated with water sources. You require to fill the demographic and responses/answer to the questions that follows.

Your responses/answers to these questions will have no direct/indirect impact on you or your family but instead will help to better understand outbreak and how to prevent water borne infections from spreading.

Your name/identity is not required and your demographic data will not be used to identify you it is strictly for this research and will be destroyed after the dissemination of the findings.

This study has no financial gain to you or the investigator, but for education purposes.

You and your family are free to choose whether or not to participate in this investigation. You are also free to say no to any part of this investigation.

There is no penalty if you or your family do not want to participate. Even if you agree to participate, you may change your mind at any time.

Kindly contact the principal investigator for any further enquiries.

**Principal Investigator:**

**Hope ONOHUEAN**

**(+256753802877).**
