# Supplementary material for: Demographic dynamics of waterborne disease and perceived associated WASH factors in Bushenyi and Sheema districts of South-Western Uganda
Source: Environ Monit Assess. 2023 Jun 20;195(7):864. doi: 10.1007/s10661-023-11270-1 (PMC10281895; doi:10.1007/s10661-023-11270-1)
Supplement: Supplementary file 3 — Supplementary file3 (DOCX 19 KB) [file 10661_2023_11270_MOESM3_ESM.docx]

(S 3) supplementary material

S 3. The linear relationship between PCA components and the knowledge and practice of WASH and water quality

| **PCA - Knowledge of WASH and Practice of WASH** | | | | | | **PCA-Water Quality** | | | | |
| --- | --- | --- | --- | --- | --- | --- | --- | --- | --- | --- |
| **Variables** | **Dim.1** | **Dim.2** | **Dim.3** | **Dim.4** | **Dim.5** | **Variables** | **Dim.1** | **Dim.2** | **Dim.3** | **Dim.4** |
| **Age** | -0.21556 | -0.17746 | 0.947436 | -0.07418 | -0.13742 | **Water borne disease** | -0.53772 | 0.548455 | 0.639256 | -0.03747 |
| **Sex** | -0.53285 | 0.338183 | 0.103195 | 0.757318 | 0.131822 | **intervention** | -0.44599 | 0.710086 | -0.53875 | 0.081368 |
| **Occupation** | -0.40643 | 0.670781 | 0.11831 | -0.41631 | 0.443843 | **Basic economic Status** | 0.837555 | 0.414647 | -0.03553 | -0.35399 |
| **Marital Status** | 0.558922 | -0.41801 | 0.133134 | 0.132313 | 0.690868 | **Water Quality** | 0.868488 | 0.304344 | 0.153392 | 0.359963 |
| **Knowledge of WASH** | 0.82037 | 0.421515 | 0.146216 | 0.055032 | -0.10688 |  |  |  |  |  |
| **Practice of WASH** | 0.844465 | 0.358108 | 0.133742 | 0.117525 | -0.09171 |  |  |  |  |  |

**Sample size determination**

Using the formula;


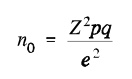
 Where: e is the desired level of precision (i.e., the margin of error), p is the (estimated) proportion of the population with the attribute of water-related illness, (410) HMIS-Health Management Information System report in Bushenyi health centre IV, and q is 1 – p.

Z =1.96, e =5%, p = 410, q = (1 – p)

n_o_ = 199
